# Case Report: A rare presentation of vascular Ehlers-Danlos syndrome with a massive hemothorax and a chest wall hematoma

**DOI:** 10.3389/fmed.2025.1648439

**Published:** 2025-08-08

**Authors:** Beisi Han, Su Li, Yutian Ye, Xiaoyu Lu, Heng Zhang

**Affiliations:** ^1^School of Medicine, Southern University of Science and Technology, Shenzhen, China; ^2^Department of Radiation Oncology, Shenzhen People’s Hospital (The First Affiliated Hospital, Southern University of Science and Technology; The Second Clinical Medical College, Jinan University), Shenzhen, China; ^3^Department of Pulmonary and Critical Care Medicine, Shenzhen Institute of Respiratory Diseases, Shenzhen People’s Hospital (The First Affiliated Hospital, Southern University of Science and Technology; The Second Clinical Medical College, Jinan University), Shenzhen, China

**Keywords:** vascular Ehlers-Danlos syndrome, hemothorax, chest wall hematoma, *COL3A1*, rare disease

## Abstract

**Background:**

Vascular Ehlers-Danlos syndrome (vEDS) is a rare but life-threatening subtype of the Ehlers-Danlos syndromes (EDS), a group of inherited connective tissue disorders with significant clinical and genetic heterogeneity. vEDS is mainly caused by mutations in the *COL3A1* gene, leading to type III collagen abnormalities. vEDS is characterized by increased vascular fragility and predisposition to serious complications such as arterial rupture and gastrointestinal perforation. However, vEDS cases with hemothorax as the primary manifestation are extremely rare and are easily misdiagnosed or underdiagnosed.

**Case presentation:**

We report a 28-year-old man who was admitted to the hospital with a sudden onset of right-sided chest and back pain. Imaging examinations and thoracentesis revealed a massive right-sided hemothorax and a right posterior chest wall hematoma. The patient had a medical history of two episodes of spontaneous pneumothorax, as well as arterial aneurysms and dissections, along with a family history of major arterial rupture. After admission, his hemoglobin level progressively declined, which stabilized following right intercostal artery embolization. Genetic testing ultimately identified a heterozygous *COL3A1* mutation, confirming the diagnosis of vEDS.

**Conclusion:**

In this case, the patient presented with a massive right-sided hemothorax and a large chest wall hematoma without any obvious precipitating factors, in the absence of other typical clinical manifestations of vEDS, such as gastrointestinal perforation, which increased the diagnostic challenge. Possible pathogenic mechanisms include type III collagen abnormalities leading to increased fragility of the subpleural vessels, triggering vascular rupture. Clinically, young patients with recurrent hemothorax or multiple arterial lesions should be kept on high alert for early genetic testing to clarify the diagnosis and optimize management. This case helps to raise awareness of the heterogeneous clinical manifestations of vEDS and to avoid misdiagnosis and underdiagnosis.

## 1 Introduction

Ehlers-Danlos syndromes (EDS) represent a heterogeneous group of inherited connective tissue disorders characterized by multisystem involvement and considerable clinical and genetic variability. Classified as a rare disease, EDS currently includes 14 distinct subtypes (1). Vascular Ehlers-Danlos syndrome (vEDS) is one of the rarer forms, with an estimated prevalence of 1:50,000–1:200,000. vEDS mainly involves mutations in the *COL3A1* or *COL1A1* genes. Most of the heterozygous mutations in the *COL3A1* gene lead to abnormalities of type III collagen, which is manifested by increased fragility of blood vessels, the gastrointestinal tract, and the uterus, and is prone to life-threatening complications, such as arterial rupture, gastrointestinal perforation, and uterine rupture in pregnancy, all of which are associated with high mortality (2).

Typical clinical manifestations of vEDS include aneurysm, arterial dissection, spontaneous sigmoid colon perforation, and skin ecchymosis (1). However, initial presentations with thoracic complications—such as spontaneous hemothorax or chest wall hematoma—are exceedingly rare and can obscure timely diagnosis. Unlike the more common cases involving abdominal or pelvic vasculature, this case features massive hemothorax and chest wall hematoma as initial symptoms, without prior visceral or major arterial rupture. This highlights a non-classical presentation that may easily be overlooked without a high index of clinical suspicion.

In this report, we describe a case of vEDS presenting with spontaneous hemothorax and a massive chest wall hematoma, and analyze the underlying genetic mechanisms. We also review relevant literature to summarize diagnostic and management strategies and discuss the clinical implications of such atypical thoracic presentations.

## 2 Case description

### 2.1 Acute presentation and initial evaluation

A 28-year-old male was admitted to our hospital on November 5, 2024, with a chief complaint of right-sided chest and back pain for over 10 h. Approximately 10 h before admission, the patient experienced a sudden onset of right-sided chest and back pain without any obvious precipitating factors, which was described as paroxysmal colic and aggravated by deep breathing. He was admitted to an outside hospital, where chest CT revealed a space-occupying lesion in the right thoracic cavity, along with pleural effusion in the right pleural cavity and interlobar fissures. After receiving analgesic treatment, he was referred to our emergency department for further evaluation. During this period, the patient gradually developed dyspnea, profuse sweating, fever, and dizziness, with SpO2 of 87% and Bp of 87/55 mmHg.

The patient’s past medical history included pulmonary bullae resection for left-sided pneumothorax in 2017, and a similar procedure for right-sided pneumothorax in 2019. Artificial vascular replacement of the right subclavian artery was performed due to a right subclavian artery aneurysm and carotid artery dissection in 2019.

Although the patient had undergone prior interventions for bilateral pneumothoraces and vascular abnormalities including subclavian artery aneurysm and iliac dissection, no formal genetic evaluation was pursued at that time. Given the absence of classical syndromic features and the emergency nature of prior presentations, the possibility of an underlying hereditary connective tissue disorder was not explicitly raised. No systematic follow-up was arranged after those earlier admissions.

In the patient’s family history, his maternal uncle died in his 40s due to abdominal aortic rupture, and his maternal grandmother died in her 50s from rupture of a major abdominal artery.

On physical examination, the patient exhibited normal development, moderate nutrition, and a normal body habitus. An old surgical scar was observed over the anterior chest. The percussion revealed dullness over the right lung, and breath sounds decreased on the right side. No obvious dry or wet rales were heard in either lung. Cardiac and abdominal examinations were unremarkable. The spine and extremities showed no deformities, with normal range of motion and muscle strength. The mobility of finger and wrist joints was normal, and skin elasticity was within normal limits.

Initial laboratory tests on November 5 revealed a white blood cell (WBC) count of 19.96 × 10^9^/L (normal range: 4–10 × 10^9^/L), a neutrophil percentage of 86.8%, a hemoglobin level of 119 g/L, and a platelet count of 365 × 10^9^/L. The interleukin-6 (IL-6) level was elevated at 16.28 pg./mL. Coagulation function was within normal limits.

**Figure 1:**
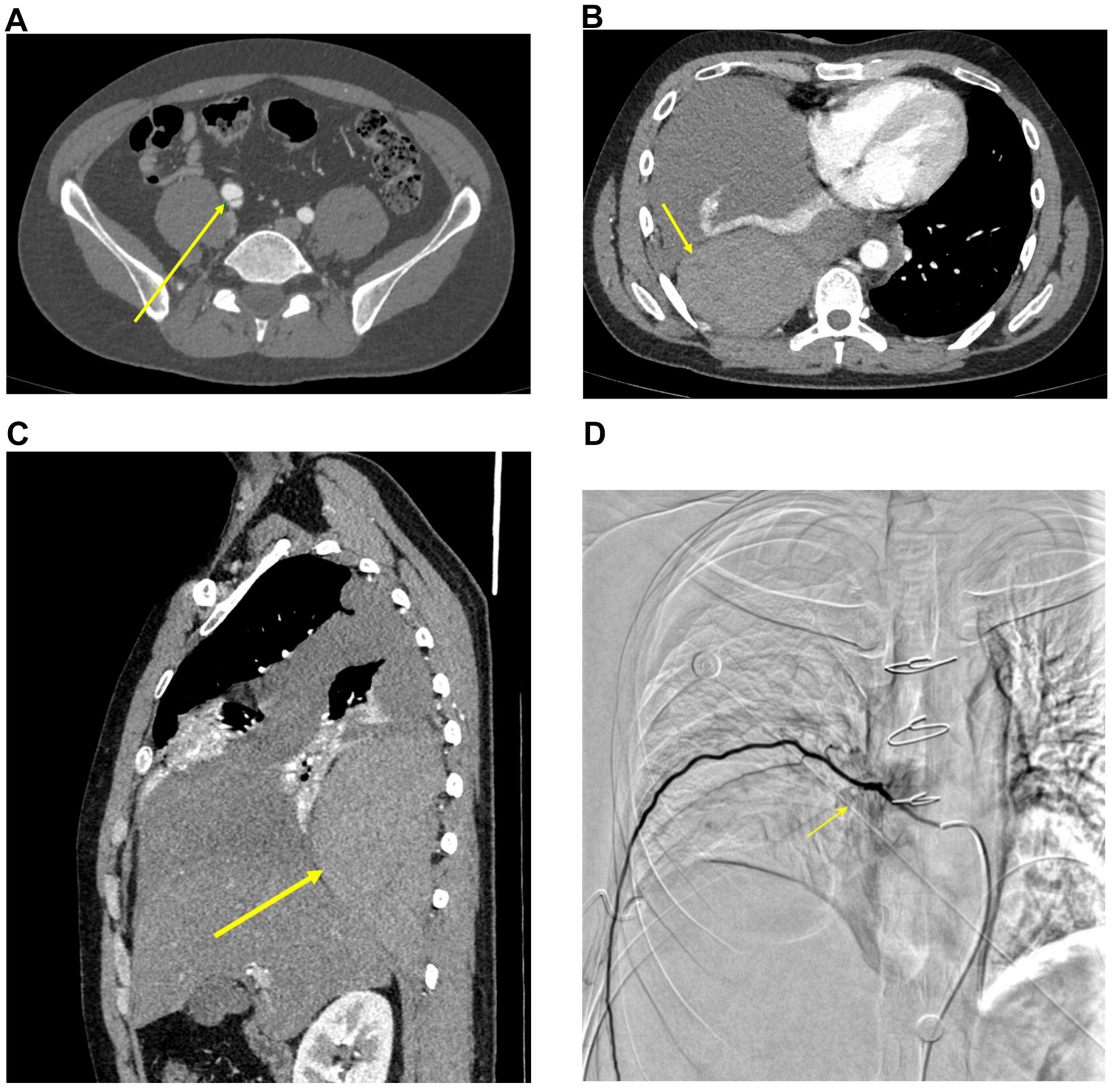
Serial imaging findings of a patient with vascular Ehlers-Danlos syndrome. **(A,B)** Axial contrast-enhanced CTA (November 5, 2024). **(A)** Right common iliac artery dissection (arrow). **(B)** Right extrapleural hematoma (arrow). **(C)** Sagittal contrast-enhanced CT (November 5, 2024), depicting the same right extrapleural hematoma (arrow). **(D)** DSA (November 8, 2024), showing percutaneous aortic and intercostal artery angiography. A dilated intercostal artery is visualized (arrow).

CT angiography (CTA) of cervical, thoracic, and abdominal arteries showed: 1. right common iliac artery dissection; 2. right extrapleural hematoma and pleural effusion, with right lung atelectasis and consolidation (Figures 1A–C).

The patient was treated with dexamethasone for anti-inflammation, ciprofloxacin for anti-infection, fluid resuscitation, and analgesia, resulting in slight relief of his chest pain.

Chest drainage was initially deferred due to imaging features suggestive of an extrapleural hematoma and clinical suspicion of underlying vascular fragility. Given the hemodynamic instability and a family history of arterial rupture, premature drainage was considered potentially hazardous before further evaluation.

### 2.2 Diagnostic workup and interventional management

On November 6, 2024 (admission day 2), blood tests showed a WBC count of 13.16 × 10^9^/L, a neutrophil percentage of 70.40%, a progressively decreasing hemoglobin level of 85 g/L (normal range: 120 ~ 160 g/L), and a platelet count of 328 × 10^9^/L. Arterial blood gas analysis on room air (FiO₂ 21%) revealed a partial pressure of oxygen (PaO₂) of 70.4 mmHg and a partial pressure of carbon dioxide (PaCO₂) of 31.7 mmHg. Autoimmune screening was negative. Tumor markers were within normal limits.

On November 8, 2024 (admission day 4), the WBC count declined to 9.36 × 10^9^/L, hemoglobin decreased to 74 g/L, and the platelet count was 343 × 10^9^/L.

Contrast-enhanced chest CT performed on the same day showed a large right extrapleural mass, suggestive of a hematoma; massive right-sided pleural effusion; extensive exudation in the right lung with incomplete expansion; mild exudation in the left lower lung; and a small amount of left-sided pleural effusion. Postoperative changes were observed in both upper lungs, along with multiple pulmonary bullae.

Under ultrasound guidance, thoracentesis was performed on the right side, draining dark red, bloody pleural fluid. Analysis of the pleural fluid showed weakly positive mucin (±), total cell counts of 3,460,070/μL, nucleated cell counts of 3,070/μL, with 68.9% polymorphonuclear cells and 31.1% mononuclear cells. The chloride concentration was 104.3 mmol/L, glucose was reduced to 3.74 mmol/L, and total protein was elevated to 50.8 g/L.

Following consultation with the interventional radiology team, the patient underwent percutaneous aortic angiography, intercostal artery angiography, and selective embolization of suspected bleeding intercostal arteries under digital subtraction angiography (DSA). No active extravasation was detected, but multiple dilated intercostal arteries were visualized (Figure 1D). Using a Cobra catheter in combination with a microcatheter, three right-sided intercostal arteries suspected of bleeding were selectively embolized with multiple coils. Post-embolization angiography confirmed slowed blood flow. The patient underwent successful embolization of three intercostal arteries, and hemoglobin levels remained stable postoperatively without further decline.

### 2.3 Genetic confirmation and follow-up

Genetic testing was performed with written informed consent and approval by the institutional ethics committee. Peripheral blood was collected in EDTA tubes for genomic DNA extraction. Whole-exome sequencing (WES) was conducted using the MGIseq-T7 platform with an internally designed whole-exome capture probe set. Sequencing reads were aligned to the UCSC hg19 reference genome, and variant calling and annotation were performed according to standard pipelines.

A heterozygous splicing variant in *COL3A1* (NM_000090.4: c.3417 + 5G > A; chr2:189872669, intron 46) was identified and classified as likely pathogenic according to ACMG/AMP and ClinGen guidelines. This variant has been previously reported to cause exon skipping (PMID: 24922459).

Sanger sequencing was performed for variant confirmation using the following primers: Forward: 5′-TCCCAGTGCTTTTTAAGGCCT-3′. Reverse: 5′-TGCTACTTACTCTGGGGCCT-3′. Product size: 116 bp; PCR annealing temperature: 60°C.

Sequencing was conducted using the BigDye Terminator v3.1 Cycle Sequencing Kit on an ABI 3730xl DNA Analyzer. Quality control was based on peak morphology, ensuring background peaks did not exceed 10% of signal height.

**Figure 2:**
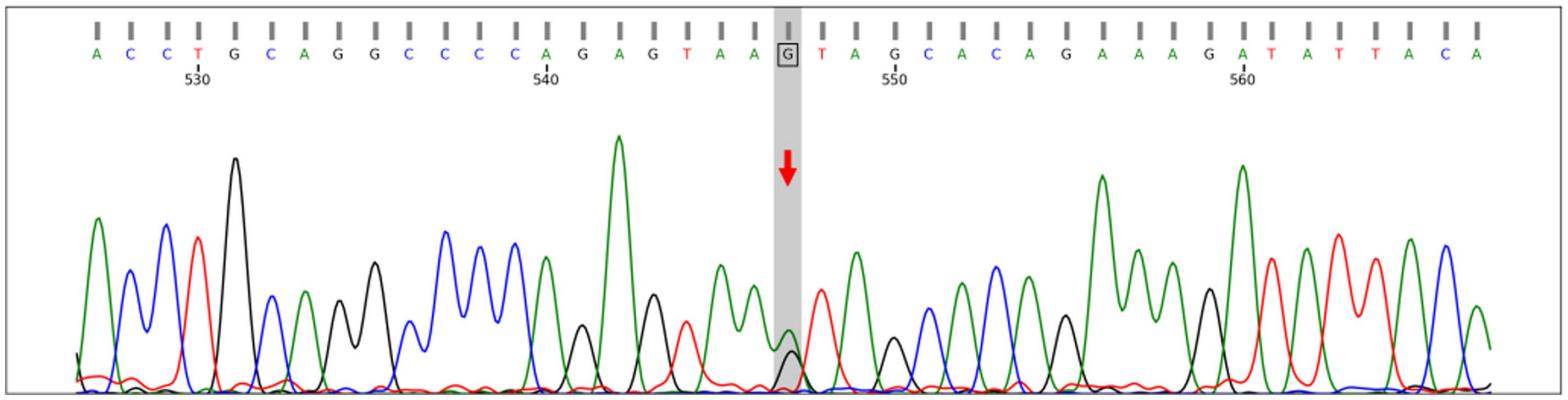
Sanger sequencing confirming the heterozygous splicing variant *COL3A1*: NM_000090.4:c.3417 + 5G > A (chr2:189872669, intron 46) in both forward and reverse directions. This variant affects the +5 position of the donor splice site and has been reported to cause exon skipping (PMID: 24922459).

This confirmed the heterozygous mutation, supporting the diagnosis of vEDS (Figure 2).

Following genetic confirmation, the patient was referred to a tertiary center for comprehensive vascular management and multidisciplinary follow-up. Prior to transfer, we provided individualized counseling and outlined a preliminary surveillance strategy based on current vEDS guidelines. This included regular vascular imaging (MRA or CTA) every 6 to 12 months to monitor for aneurysm formation or dissection, along with strict blood pressure control. *β*-blocker therapy was recommended to reduce arterial wall stress. Antiplatelet and statin therapies were not initiated, as there were no clear indications.

The patient was also informed of the autosomal dominant inheritance pattern of vEDS, with a 50% chance of transmission to offspring. Genetic counseling was provided, and first-degree relatives were advised to undergo clinical evaluation and genetic testing where appropriate.

**Figure 3:**
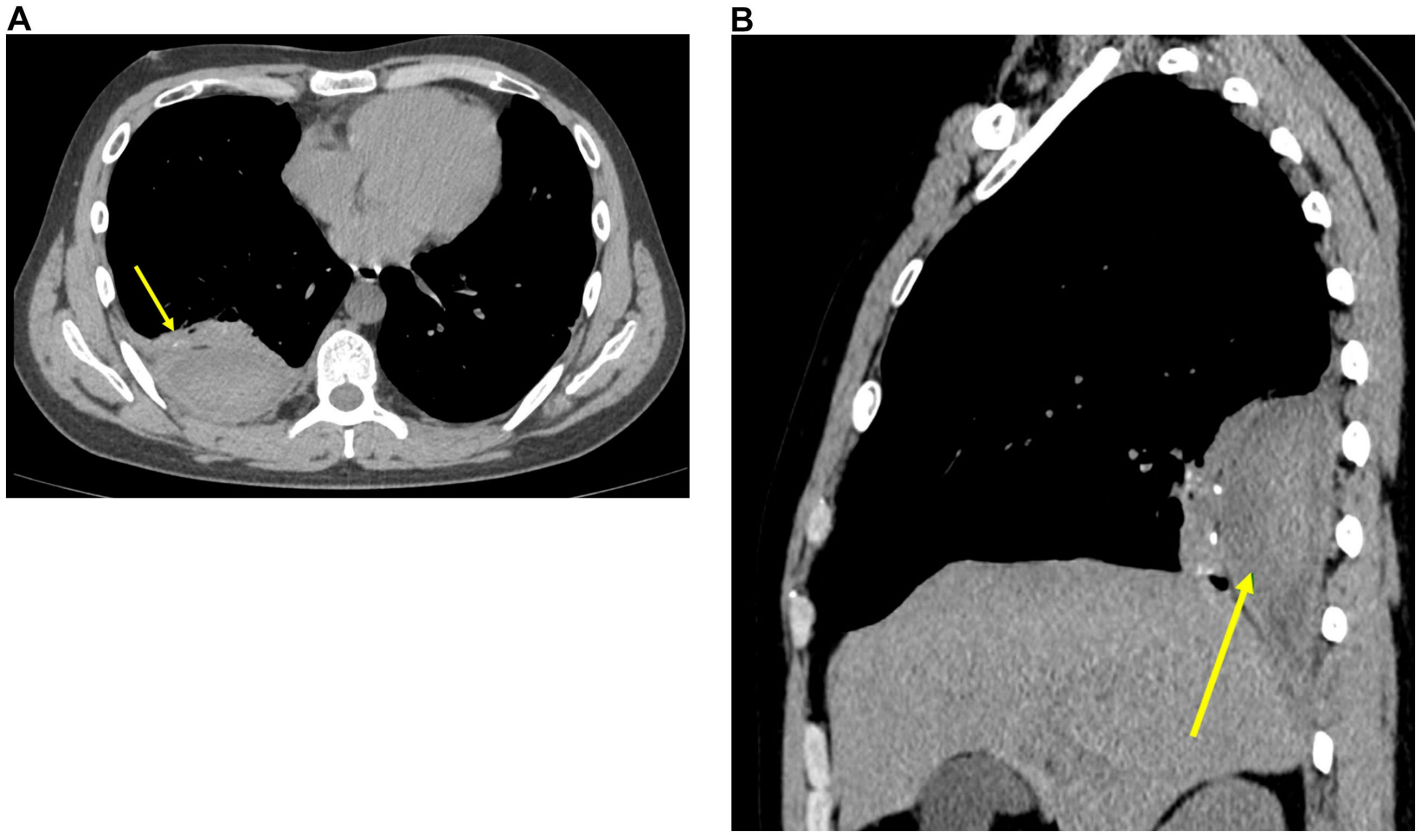
Follow-up chest CT (December 27, 2024). **(A)** Axial non-contrast CT shows a reduction in the size of the right extrapleural hematoma (arrow), with marked decrease in right pleural effusion and resolution of right lower lobe atelectasis. **(B)** Sagittal non-contrast CT demonstrating the residual right extrapleural hematoma (arrow).

On December 27, 2024, follow-up chest CT demonstrated postoperative changes in the right lower lobe of the lung. The previously noted posterior right chest wall hematoma had decreased in size compared to prior imaging. Additionally, the large right pleural effusion was markedly reduced (Figures 3A,B).

A clinical timeline summary is provided in Table 1.

**Table 1:** Chronology of clinical events.

Date	Hospital day	Clinical event
Nov 5, 2024	Day 1	Sudden right-sided chest pain; initial CT at outside hospital showing thoracic lesion and pleural effusion. Transferred to our hospital. Baseline labs and CTA performed.
Nov 6, 2024	Day 2	Hemoglobin dropped to 85 g/L; autoimmune and tumor markers negative.
Nov 8, 2024	Day 4	Worsening pleural effusion; chest CT showed extrapleural hematoma. Thoracentesis performed. Intercostal artery embolization via DSA completed.
Nov 30, 2024	/	Whole-exome sequencing confirmed COL3A1 c.3417 + 5G > A mutation. Diagnosis of vEDS established.
Dec 27, 2024	Follow-up	Chest CT showed reduction in hematoma and pleural effusion. No new complications noted.

## 3 Discussion

vEDS is a rare inherited connective tissue disorder, with an estimated prevalence of 1:50,000 to 1:200,000. It involves mutations in the *COL3A1* and COL1A1 genes, with most cases attributed to *COL3A1* heterozygous mutations, leading to abnormalities in type III collagen (2). Although vEDS follows an autosomal dominant inheritance pattern, only approximately 38% of patients have a positive family history, and about 50% of *COL3A1* mutations are *de novo* mutations (3, 4).

The *COL3A1* gene encodes the *α*-chain of type III collagen, a crucial structural protein distributed extensively in connective tissues such as the skin, lungs, blood vessels, and gastrointestinal tract (5). Mutations in *COL3A1* disrupt glycine residues within the collagen triple helix, impairing its stability and weakening the strength and elasticity of connective tissues (6). These defects in collagen synthesis directly contribute to the increased vulnerability of blood vessels and internal organs in vEDS patients, leading to life-threatening complications such as arterial rupture, dissection, and visceral perforations (4).

The clinical manifestations of vEDS are highly heterogeneous and are classified into major and minor criteria. Major features include arterial rupture, spontaneous sigmoid colon perforation, late-pregnancy uterine rupture or severe perineal laceration after delivery, and carotid-cavernous sinus fistula (2, 7). Arterial lesions most commonly involve abdominal aortic branches and aortic branches; pulmonary vascular involvement is exceedingly rare (8, 9). In addition, vEDS patients may present with 12 recognized minor features, including spontaneous pneumothorax and skin or joint abnormalities (2). Due to symptom overlap with other connective tissue disorders and vascular diseases, diagnosing vEDS remains a clinical challenge.

Before genetic confirmation, other heritable connective tissue disorders were considered in the differential diagnosis, particularly Marfan syndrome and Loeys-Dietz syndrome. These conditions also involve vascular fragility, aortic aneurysms, and dissection. However, the absence of features such as ectopia lentis, long bone overgrowth, or craniofacial dysmorphisms made Marfan syndrome less likely. Loeys-Dietz syndrome was considered due to the presence of arterial tortuosity and aneurysms, but the patient lacked characteristic features such as hypertelorism, bifid uvula, or cleft palate. Negative autoimmune screening and the presence of a COL3A1 splicing variant ultimately supported the diagnosis of vEDS.

In the present case, the patient presented with a massive hemothorax and a right-sided chest wall hematoma, which is atypical compared to the commonly reported initial manifestations of vEDS. These features complicated the clinical diagnosis. However, the combination of a suggestive family history, repeated spontaneous pneumothoraces, and multiple arterial lesions raised the suspicion of vEDS. The patient, aged 28 years, had a family history of two relatives who died prematurely due to arterial rupture, strongly suggesting an underlying hereditary disorder. In addition, the patient had a history of hemoptysis and developed right iliac artery dissection, right subclavian artery aneurysm, and carotid artery dissection, indicating systemic multiple arterial abnormalities. Notably, the occurrence of hemothorax in this case was accompanied by multiple pulmonary bullae predominantly in the upper lobes, despite the patient having no history of smoking. This unusual presentation suggests that underlying connective tissue abnormalities may have contributed to increased fragility of the subpleural vasculature, thereby precipitating the hemothorax. Typical vEDS features, such as characteristic skin or limb findings, were absent in this case.

Epidemiological data indicated that 25% of patients with vEDS manifested prominent symptoms before the age of 20, and over 80% experienced disease onset before the age of 40. The reported median survival was 48 years (3). The most common initial manifestation was arterial rupture (46%) or gastrointestinal perforation (19%), while the incidence of pneumothorax or hemothorax (P/HTX) was relatively low, at about 12.5 and 3%, respectively (10). Nevertheless, some studies have reported that spontaneous P/HTX may precede arterial lesions in 17.7% of vEDS patients (11).

A comparable case was reported by Dar et al. in 2012, describing a 21-year-old male who presented with spontaneous hemo-pneumothorax and was later diagnosed with vEDS (12). Notably, this patient had no family history or known vascular lesions. Moreover, while thoracotomy was performed in that case, our patient was managed non-surgically with intercostal artery embolization. To our knowledge, this is one of the few reported cases of vEDS presenting with both hemothorax and chest wall hematoma, confirmed by CTA/DSA and successfully treated with embolization, highlighting its diagnostic and therapeutic significance.

The potential mechanism of hemothorax and chest wall hematoma in vEDS may involve abnormalities of type III collagen leading to increased fragility of the subpleural vessels and intercostal arteries, resulting in spontaneous hemorrhage. When the bleeding is confined to the parietal pleura, it may accumulate to form a large chest wall hematoma when confined by the parietal pleura, which accounted for the sudden onset of severe chest pain in this patient. In contrast, when the bleeding is not contained by the pleural structures and directly enters the pleural cavity, it results in hemothorax. The diagnosis of vEDS relies on a combination of clinical presentation and genetic testing. Mutations in the *COL3A1* gene are the primary pathogenic cause of vEDS, with common mutation types including glycine substitutions, splice-site mutations, and nonsense mutations (13). In this case, genetic analysis identified a NM_000090.4:c.3417 + 5G > A heterozygous mutation in the *COL3A1* gene, located near a splice site. Bioinformatic analysis predicted this variant to be likely pathogenic, potentially leading to defective collagen synthesis and an increased risk of arterial rupture.

Currently, there is no curative treatment for vEDS (14). Therefore, early recognition and diagnosis are critical for improving patient prognosis. Studies have shown that spontaneous P/HTX in vEDS patients often precedes arterial or gastrointestinal complications (11). Therefore, in young patients presenting with spontaneous P/HTX, vEDS should be considered as part of the differential diagnosis, and other vEDS-related features should be carefully evaluated for early diagnosis and timely intervention. In any young patient presenting with spontaneous hemothorax, especially with family history or prior pneumothorax, vEDS should be considered and prompt genetic evaluation initiated.

Once vEDS is diagnosed, an individualized management strategy should be developed to reduce the risk of vascular complications. Regular imaging surveillance, including CTA and MRA, can assist in the early detection of vascular abnormalities, such as aneurysms and arterial dissections, allowing for timely intervention to prevent further complications (15). In addition, patients should avoid activities that may trigger arterial injury, such as frictional trauma, strenuous exercise, and unnecessary surgical procedures (16). Control of blood pressure and heart rate is also crucial to reduce stress on the arterial walls and the risk of vascular rupture. Studies have suggested that *β*-blockers, such as propranolol, may help lower hemodynamic load and potentially decrease the incidence of vascular events, although further clinical evidence is needed (17).

For vEDS patients who develop arterial rupture, aneurysm, or dissection, endovascular intervention can be considered the preferred approach to reduce the risk of fatal arterial complications (18). In this case, the hemothorax was effectively controlled by intercostal artery embolization, preventing further blood loss. Genetic counseling is of great importance for vEDS patients and their family members. Genetic testing can help identify other potential carriers within the family, enabling early intervention and improving prognosis. Given that some vEDS patients lack the characteristic skin or facial features, and that approximately 50% of cases are caused by *de novo* mutations, a high level of clinical vigilance is required, and genetic testing should be actively considered for definitive diagnosis in suspicious cases (19).

In addition to early diagnosis and genetic counseling, long-term management plays a critical role in improving outcomes for patients with vEDS. Regular vascular imaging—typically via CTA or MRA at 6- to 12-month intervals—is advised to detect aneurysm formation, progression, or dissection at an early stage (15). Furthermore, Multidisciplinary care involving vascular surgery, cardiology, clinical genetics, and radiology is recommended to address the multisystemic nature of vEDS (20). In selected patients, psychological support and nutritional guidance may also contribute to improved quality of life. These comprehensive strategies emphasize the need for individualized and proactive care in vEDS (20).

## 4 Conclusion

This is a case of vEDS with a massive hemothorax and chest wall hematoma. The initial manifestation of this case was significantly different from the typical features of vEDS reported in the literature, such as arterial rupture or gastrointestinal perforation, making the diagnosis more challenging. Clinically, in young patients presenting with spontaneous hemothorax or chest wall hematoma, particularly when accompanied by arterial abnormalities such as dissection or pulmonary bullae in non-smokers, a detailed family history should be obtained, and genetic testing should be considered as early as possible to establish a definitive diagnosis. This case highlights the diverse clinical manifestations of vEDS and underscores the importance of improving awareness to prevent misdiagnosis and missed diagnosis.

## Data Availability

The original contributions presented in the study are included in the article/supplementary material, further inquiries can be directed to the corresponding author.
